# Development and validation of a prediction model for hypernatremia in patients with severe burns

**DOI:** 10.3389/fmed.2026.1963702

**Published:** 2026-09-30

**Authors:** Bo Liu, Weixia Cai, Luyang Zhao, Xiaolong Hu, Peng Ji, Chenyang Tian, Feifei Deng, Jiaqi Liu, Zhao Zheng

**Affiliations:** 1 Department of Burns and Cutaneous Surgery, Xijing Hospital, Fourth Military Medical University, Xi'an, China; 2 Department of Wuhua Branch Outpatient, 920th Hospital of PLA Joint Logistics Support Force, Kunming, China

**Keywords:** hypernatremia, severe burns, nomogram, prediction model, risk stratification, web-based calculator

## Abstract

Hypernatremia is a prevalent and serious electrolyte disturbance in patients with severe burns, associated with significantly increased mortality and prolonged ICU stays. Early identification of high-risk patients could enable timely preventive interventions; however, practical tools for individualized risk stratification are currently lacking. This study aimed to develop and validate a nomogram and a web-based calculator for the early prediction of hypernatremia in patients with severe burns. A total of 592 patients with burns covering ≥30% total body surface area were retrospectively included and randomly assigned to a training cohort (*n* = 415) and an internal validation cohort (*n* = 177). Six independent predictors were identified: TBSA, mechanical ventilation, blood urea nitrogen ≥10 mmol/L, sepsis, glucose ≥9 mmol/L, and inhalation injury. The nomogram achieved an apparent C-index of 0.929 and a bootstrap-corrected C-index of 0.923 in the training cohort, and a C-index of 0.953 in the internal validation cohort. Using dual cutoffs of 90 and 135 points, the low cutoff yielded negative predictive values of 96.4% and 98.1%, and the high cutoff yielded positive predictive values of 75.4% and 65.3% in the training and internal validation cohorts, respectively. The nomogram and web-based calculator provide accurate, individualized risk prediction, facilitating early identification of high-risk individuals and supporting preventive strategies.

## 1 Introduction

Severe burns, caused by thermal, chemical, or electrical insults, are devastating injuries characterized by highly complex pathophysiological changes. Hypernatremia (serum sodium >145 mmol/L) is one of the most common electrolyte disorders in this population, with reported incidences ranging from 9.9% to 24.4% (1). Its occurrence reflects disease severity and profound disturbances of internal homeostasis, and it is associated with worse clinical outcomes and higher mortality (1–3).

At present, hypernatremia is diagnosed solely by laboratory measurement of serum sodium. However, by the time serum sodium levels are markedly elevated, patients often already exhibit severe intracellular dehydration and neuropsychiatric manifestations, which substantially complicate treatment (4, 5). Early prediction of hypernatremia risk is therefore of considerable clinical importance. Identifying high-risk patients in advance would enable clinicians to implement preemptive measures—such as refined fluid resuscitation strategies, adjustment of diuretic use, and avoidance of iatrogenic sodium loading—to potentially lower the incidence of hypernatremia (6). In addition, patients recognized as high-risk constitute a critical target group for intensified monitoring and individualized fluid management (7).

Over the past several years, some progress has been made in predicting burn-related electrolyte disturbances. Several studies have reported that TBSA and full-thickness burn area are strongly associated with the development of hypernatremia (8). However, other investigators have found that clinical characteristics alone have limited predictive value and that incorporating additional laboratory biomarkers is necessary to improve accuracy (9). Serum biomarkers such as interleukin−6 and procalcitonin are thought to reflect the intensity of the post-burn inflammatory response and may be useful for prediction (8), although these markers can also be markedly elevated in burn patients without hypernatremia. Moreover, although several risk scoring systems have been proposed for prognostic assessment in burn patients, their value for predicting hypernatremia risk remains unclear (10, 11).

In the absence of a specific and practical predictive method, there is a clear need for a comprehensive prediction model that integrates both clinical and laboratory data. Among available tools, nomograms translate complex regression models into visual diagrams that provide individualized, intuitive, and relatively accurate risk estimates. Nomograms are simple to apply at the bedside and can assist clinicians in real-time decision-making. To our knowledge, this is the first nomogram-based prediction model developed specifically to estimate the risk of hypernatremia in patients with severe burns.

## 2 Methods

### 2.1 Study population

We retrospectively collected data from consecutive patients with severe burns admitted to the Burns and Cutaneous Surgery Intensive Care Unit of Xijing Hospital, Air Force Medical University, between January 2016 and December 2025. The study was approved by the Institutional Ethics Committee of Xijing Hospital, and the requirement for informed consent was waived because of its retrospective design.

Inclusion criteria were: (1) age ≥18 and ≤75 years; (2) TBSA ≥30%; (3) admission within 48 h after injury; and (4) hospital length of stay ≥72 h. Exclusion criteria were: (1) preexisting hypernatremia at admission (serum sodium >145 mmol/L); (2) severe traumatic brain injury (Glasgow Coma Scale ≤8 at admission or intracranial hemorrhage requiring neurosurgical intervention) or other major trauma (Injury Severity Score >15 or any trauma requiring immediate non-burn surgical intervention), as these conditions can independently disrupt fluid and electrolyte balance. In cases of severe traumatic brain injury, central diabetes insipidus may also develop; (3) history of severe renal insufficiency (serum creatinine >177 μmol/L before admission), since preexisting renal dysfunction directly impairs sodium and water regulation; (4) history of diabetes mellitus with poor glycemic control (HbA1c > 8% or insulin-dependent diabetes with documented poor adherence), as chronic hyperglycemia can lead to osmotic diuresis; (5) pregnancy or lactation, because physiological changes during these periods can alter fluid and electrolyte homeostasis, and (6) substantially incomplete clinical data (missing key variables >30%). Eligible patients were randomly assigned, in a 7:3 ratio, to a training cohort (*n* = 415) for nomogram development and an internal validation cohort (*n* = 177) for performance assessment.

### 2.2 Clinical management and data collection

All patients received standardized care according to our institutional burn management protocols. Fluid resuscitation during the first 24 h after injury was guided by the modified Parkland formula and was dynamically adjusted according to urine output, heart rate, blood pressure, and other clinical parameters (12). Early eschar excision and skin grafting were performed to promote timely wound closure. Additional comprehensive care, including antibiotic therapy, nutritional support, and organ function support, was provided as clinically indicated.

Routinely collected clinical data included demographic variables (age, sex); burn characteristics (TBSA, full-thickness burn area, presence of inhalation injury); laboratory measurements (maximum blood glucose within 24 h of admission, minimum serum albumin within 72 h, maximum BUN within 72 h, admission serum creatinine, admission interleukin−6, admission procalcitonin, admission white blood cell count and neutrophil count); treatment-related variables (mechanical ventilation, airway incision, use of a fluidized bed); and complications (acute kidney injury, sepsis). To facilitate early prediction using the model, all predictor variables were evaluated during the initial post-admission period, prior to the first documented episode of hypernatremia. Mechanical ventilation and sepsis were noted based on their occurrence at admission or within the first 48 h of hospitalization. Laboratory parameters were collected within the first 24–72 h, as previously indicated. Hypernatremia was defined as at least two serum sodium measurements >145 mmol/L during hospitalization. The primary endpoint was the development of hypernatremia during hospitalization; secondary endpoints were 90-day all-cause mortality and ICU length of stay.

### 2.3 Clinical variables

The clinical variables for this study are summarized in Table 1. TBSA and full-thickness burn area were evaluated and documented by the attending physician using the Rule of Nines and the Lund–Browder chart (13). Inhalation injury was diagnosed based on clinical history (enclosed-space burn, facial burns, etc.), bronchoscopic findings, and/or carboxyhemoglobin levels (14–16). Acute kidney injury was identified according to the Kidney Disease: Improving Global Outcomes (KDIGO) criteria (17). Sepsis was diagnosed according to the American Burn Association consensus criteria for sepsis and infection in burn patients (18). All laboratory tests were conducted in the Department of Laboratory Medicine at Xijing Hospital following standard procedures.

**Table 1:** Participant characteristics.

Variable	Cohort, No. (%)		*P* value
	Training (*n* = 415)	Validation (*n* = 177)	
Age, mean (SD), y	41.3 (13.4)	39.2 (14.3)	.09
Sex			
Male	333 (80.2)	131 (74.0)	.10
Female	82 (19.8)	46 (26.0)	
TBSA, mean (SD), %	57.0 (23.3)	54.7 (24.4)	.28
Full-thickness burn area			
＞ 20%	215 (51.8)	83 (46.9)	.28
≤ 20%	200 (48.2)	94 (53.1)	
IL−6, pg/mL			
＞ 200	115 (27.7)	48 (27.1)	.92
≦ 200	300 (72.3)	129 (72.9)	
PCT, ng/mL			
＞ 0.5	176 (42.4)	68 (38.4)	.41
≤ 0.5	239 (57.6)	109 (61.6)	
Minimum Albumin, mean (SD), g/L	26.9 (5.4)	27.4 (5.9)	.28
WBC, mean (SD), ×10⁹/L	20.4 (10.5)	20.6 (11.5)	.82
NEUT, mean (SD), ×10⁹/L	17.5 (9.7)	17.6 (10.3)	.85
Mechanical Ventilation			
Yes	145 (34.9)	55 (31.1)	.39
No	270 (65.1)	122 (68.9)	
Airway Incision			
Yes	159 (38.3)	56 (31.6)	.14
No	256 (61.7)	121 (68.4)	
Cr, mean (SD), μmol/L	88.0 (56.8)	93.4 (77.2)	.41
BUN, mean (SD), mmol/L	9.3 (6.1)	9.7 (7.6)	.59
Sepsis			
Yes	45 (10.8)	17 (9.6)	.77
No	370 (89.2)	160 (90.4)	
Glucose, mean (SD), mmol/L	8.4 (3.9)	8.0 (3.4)	.24
Inhalation Injury			
Yes	202 (48.7)	75 (42.4)	.18
No	213 (51.3)	102 (57.6)	
Fluidized Bed			
Yes	153 (36.9)	54 (30.5)	.16
No	262 (63.1)	123 (69.5)	

BUN, blood urea nitrogen; Cr, creatinine; IL−6, interleukin 6; NEUT, neutrophil count; PCT, procalcitonin; TBSA, total body surface area; WBC, white blood cell count.

### 2.4 Statistical analysis

Continuous variables are reported as mean (SD) or median [interquartile range (IQR)] and were compared using the unpaired *t*-test or the Mann–Whitney *U* test, as appropriate. Categorical variables are presented as number (percentage) and were compared using the *χ*^2^ test or Fisher's exact test. Survival curves were estimated with the Kaplan–Meier method and compared using the log-rank test. To account for the competing risk of death when analyzing ICU discharge, a Fine-Gray subdistribution hazard model was used. ICU discharge was treated as the event of interest, while death in the ICU served as the competing event. To address the time-dependent nature of hypernatremia in survival comparisons, a landmark analysis was conducted at day 7. Patients who were still in the ICU at day 7 were classified based on whether hypernatremia had developed by that time. Subsequent 90-day survival was then compared between these groups using a Cox proportional hazards model, starting from the landmark time point. Patients with >30% missing data for key variables were excluded. For the remaining patients, missing data were minimal (<5%) and were handled using complete-case analysis.

The training and internal validation cohorts were created using a random 7:3 split. This ratio was selected to ensure a sufficiently large training sample for stable model development, while also maintaining an independent hold-out cohort for internal validation. Although splitting the sample reduced the size of the training cohort compared to using the entire dataset, this limitation was addressed by applying internal validation with 1,000 bootstrap resamples and 10-fold cross-validation. The apparent, bootstrap-corrected, and cross-validated C-index values were consistent (0.929, 0.923, and 0.924, respectively), indicating that the model remained robust despite the split-sample approach.

Univariable logistic regression was used to assess the association between each variable and the occurrence of hypernatremia in the training cohort. In the univariable analysis, all continuous variables were entered in their original scale. Variables with *P* < 0.10 in the univariable analysis were considered candidates for multivariable logistic regression. The initial multivariable model included all continuous candidate variables and was fitted using backward stepwise selection, with a removal criterion of *P* ≥ 0.05, to identify independent risk factors. The continuous predictors selected in this step (BUN and Glucose) were then dichotomized according to receiver operating characteristic (ROC) curve analysis (maximizing the Youden index) and clinically relevant cutoffs (BUN ≥10 mmol/L vs. < 10 mmol/L; Glucose ≥9 mmol/L vs. < 9 mmol/L). TBSA was retained as a continuous variable, and mechanical ventilation, sepsis, and inhalation injury were kept as binary variables. The final model was evaluated for overall significance using the likelihood ratio test, and the significance of individual coefficients was tested with the Wald test. Effect sizes were reported as odds ratios (ORs) with 95% confidence intervals (CIs). Model performance was assessed using the Nagelkerke R^2^ and the Brier score.

Using the final multivariable logistic regression model, we constructed a nomogram with the rms package (version 8.1.1) in R and developed a web-based interactive calculator with the shiny package, deployed on the shinyapps.io platform for clinical use. The nomogram was generated by proportionally converting each variable's regression coefficient and observed range into a 0–100 point scale, with the variable contributing the most to the predicted probability assigned 100 points. The total score, obtained by summing the points for all variables, was then mapped to the corresponding predicted probability. Model discrimination was quantified using the C-index (concordance index), and its 95% CI was estimated with DeLong's method. Model calibration was examined using calibration plots. Internal validation was conducted with 1,000 bootstrap resamples to estimate optimism and obtain an optimism-corrected C-index. To further evaluate model robustness, we performed 10-fold cross-validation, randomly partitioning the training cohort into 10 equal subsets; in each iteration, nine subsets were used for model fitting and one for validation. The mean and standard deviation of the C-index across the 10 folds were reported.

To further refine risk stratification, we performed ROC curve analysis to identify two cutoff values: a low cutoff that maximized the negative predictive value (NPV) to rule out low-risk patients, and a high cutoff that maximized the positive predictive value (PPV) to rule in high-risk patients. Based on these cutoffs, patients were categorized into low-, intermediate-, and high-risk groups. The performance of each cutoff was evaluated by sensitivity, specificity, PPV, NPV, and likelihood ratios, and 95% CIs were calculated using the Wilson method.

In all analyses, *P* < 0.05 was considered statistically significant. All statistical analyses were conducted using SPSS version 26.0 (IBM Corp.) and R software version 4.6.0.

## 3 Results

### 3.1 Clinicopathologic characteristics

During the study period, 743 consecutive patients with severe burns were hospitalized. Of these, 592 patients met the inclusion criteria and were enrolled, with 415 and 177 patients assigned to the training and internal validation cohorts, respectively (Figure 1). The clinicopathologic characteristics of these patients are summarized in Table 1. Baseline demographic and clinical variables were similar between the two cohorts. Hypernatremia was observed in 113 of 415 patients (27.2%) in the training cohort and 38 of 177 patients (21.5%) in the internal validation cohort. A comparison of clinical characteristics between patients with and without hypernatremia is provided in Supplementary Table S1. Among the 151 patients who developed hypernatremia, the median time from admission to the first episode was 5 days (IQR, 2–7), and the median peak serum sodium level was 156.4 mmol/L (IQR, 149.5–163.4).

**Figure 1:**
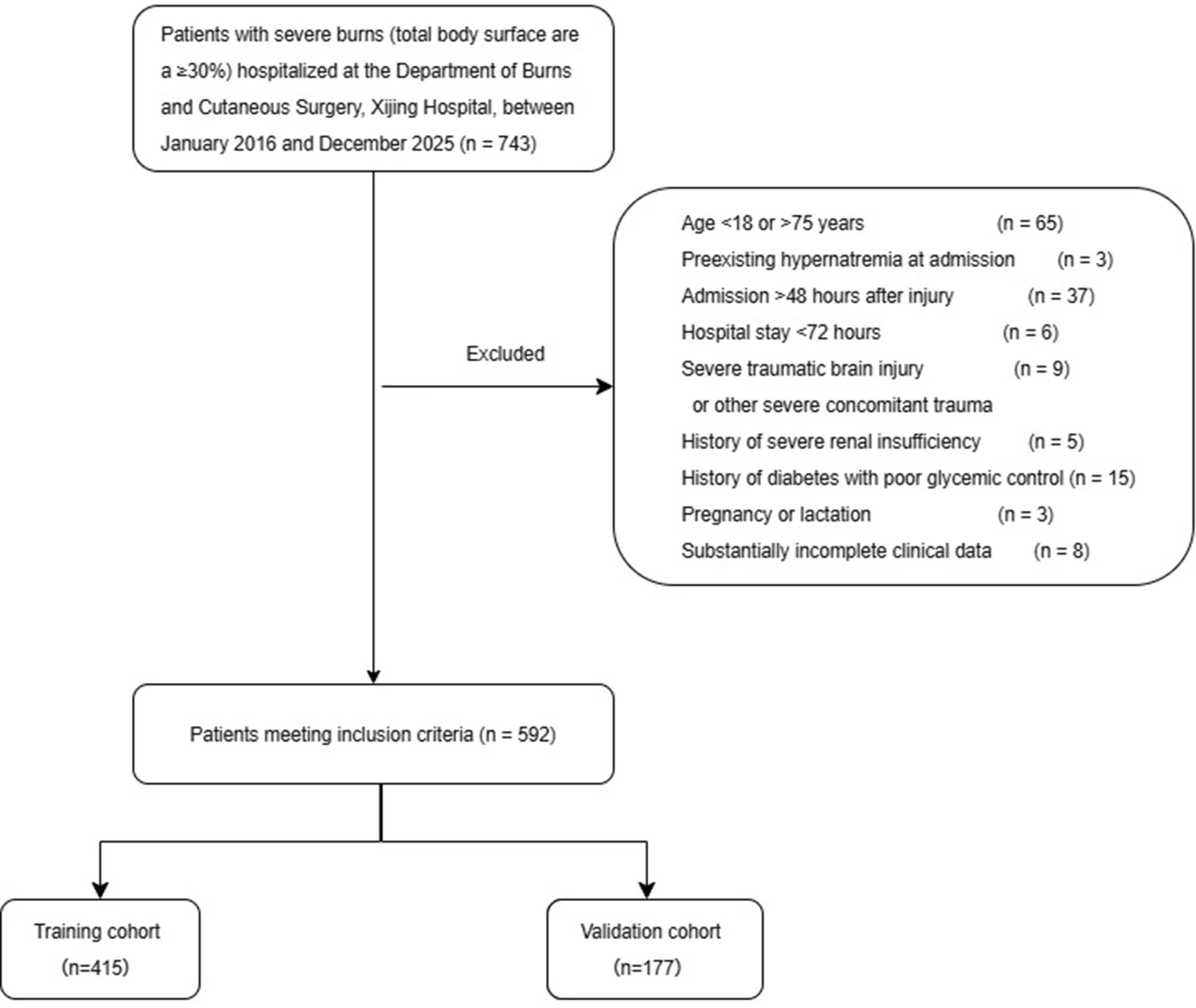
Flow chart of the study.

### 3.2 Prognostic implications of hypernatremia

The study was censored on December 31, 2025. In the training cohort, ninety-day mortality was 39.8% among patients with hypernatremia, who had a median ICU length of stay of 48 days (IQR, 22–85 days). In contrast, patients without hypernatremia had a ninety-day mortality of 1.3% and a median ICU length of stay of 18.5 days (IQR, 7–40 days) (*P* < 0.001 for both comparisons). In the internal validation cohort, ninety-day mortality was 34.2% in patients with hypernatremia and 5.0% in those without hypernatremia (*P* < 0.001). The median ICU length of stay was 40 days (IQR, 21.5–62.75 days) for patients with hypernatremia and 17 days (IQR, 9–33 days) for those without (*P* < 0.001). Kaplan–Meier curves showed that patients with hypernatremia had significantly lower probabilities of ICU discharge and ninety-day survival than those without hypernatremia (log-rank *P* < 0.001) (Figure 2).

**Figure 2:**
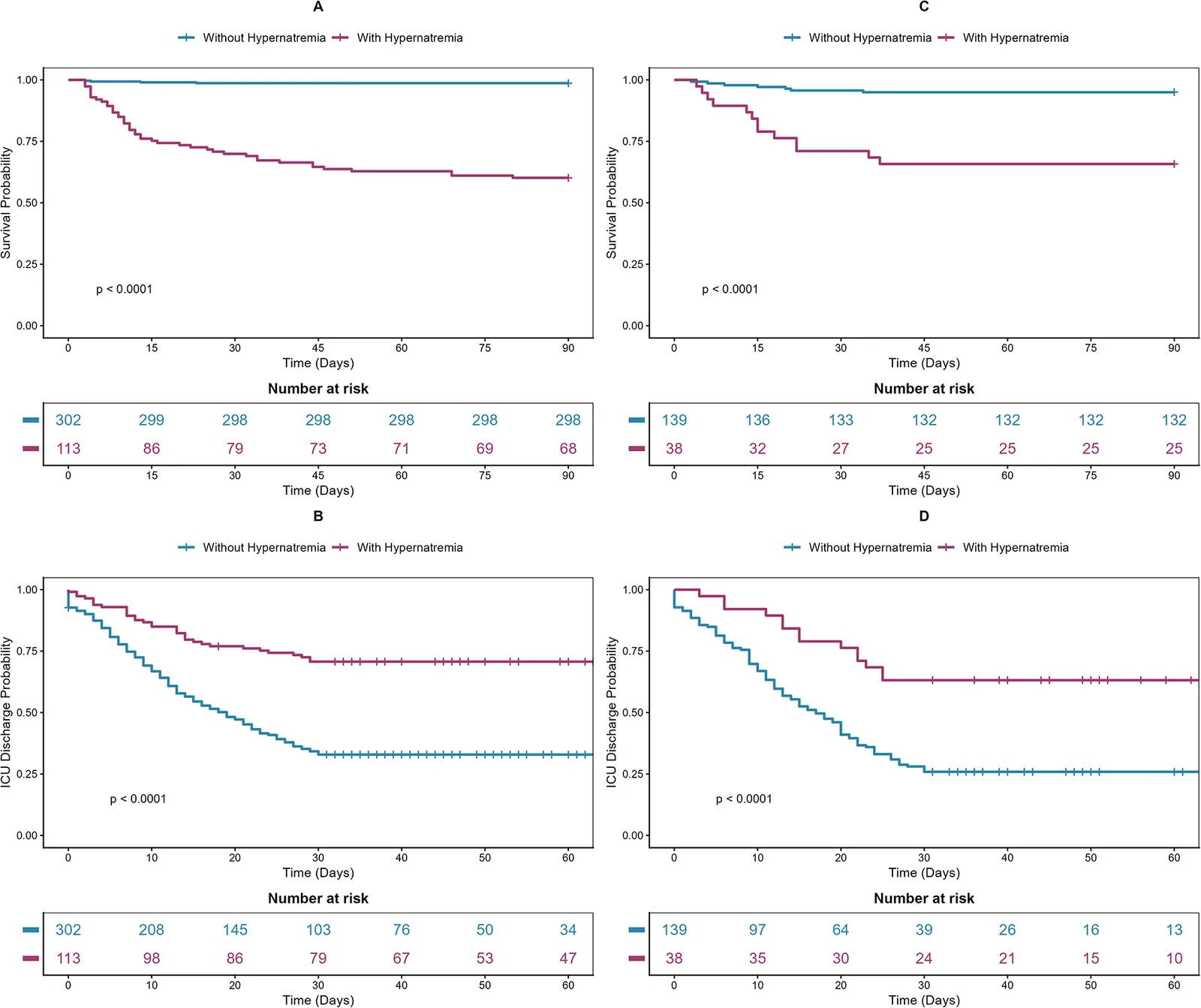
90-day survival and ICU discharge rate based on hypernatremia status. **(A,B)** 90-day survival and ICU discharge rate in the training cohort (both *P* < 0.001). **(C,D)** 90-day survival and ICU discharge rate in the internal validation cohort (both *P* < 0.001). ICU, intensive care unit.

To account for the competing risk of death, a Fine-Gray subdistribution hazard model was used. In this analysis, hypernatremia was linked to a significantly lower probability of ICU discharge in both the training cohort [subdistribution hazard ratio (sHR), 0.32; 95% CI, 0.22–0.47; *P* < 0.001] and the internal validation cohort (sHR, 0.34; 95% CI, 0.20–0.59; *P* < 0.001). To address the time-dependent nature of hypernatremia, a landmark analysis was conducted at day 7. Patients who developed hypernatremia by day 7 had significantly worse subsequent 90-day survival compared to those who did not. This finding was consistent in both the training cohort [hazard ratio (HR), 6.36; 95% CI, 3.11–13.02; *P* < 0.001] and the internal validation cohort (HR, 3.12; 95% CI, 1.08–9.00; *P* = 0.035).

### 3.3 Development and validation of the hypernatremia-predicting nomogram

All variables included in this analysis were derived from data collected in the early post-admission period. Burn-related variables (including TBSA, full-thickness burn area, and inhalation injury) were assessed and documented by the attending physician at admission.

The results of the univariable logistic regression analysis are shown in Table 2. Multivariable analysis was first conducted using continuous variables and backward stepwise selection. Based on ROC curve analysis and clinically relevant cutoffs, BUN and Glucose were then dichotomized (BUN ≥10 mmol/L vs. < 10 mmol/L; Glucose ≥9 mmol/L vs. < 9 mmol/L). TBSA was retained as a continuous variable, and the remaining predictors were kept in their original binary form. The final model included six independent risk factors (Table 3): larger TBSA (per 1% increase, OR, 1.043; 95% CI, 1.025–1.061; *P* < 0.001), mechanical ventilation (OR, 4.38; 95% CI, 2.20–8.73; *P* < 0.001), BUN ≥10 mmol/L (OR, 4.79; 95% CI, 2.42–9.49; *P* < 0.001), sepsis (OR, 6.22; 95% CI, 1.88–20.58; *P* = 0.003), Glucose ≥9 mmol/L (OR, 2.01; 95% CI, 1.02–3.96; *P* = 0.045), and inhalation injury (OR, 2.06; 95% CI, 1.02–4.16; *P* = 0.045). This variable transformation yielded a more concise and user-friendly nomogram while preserving high predictive performance. These six independent risk factors were used to construct the hypernatremia risk prediction nomogram (Figure 3A) and the web-based interactive calculator.

**Figure 3:**
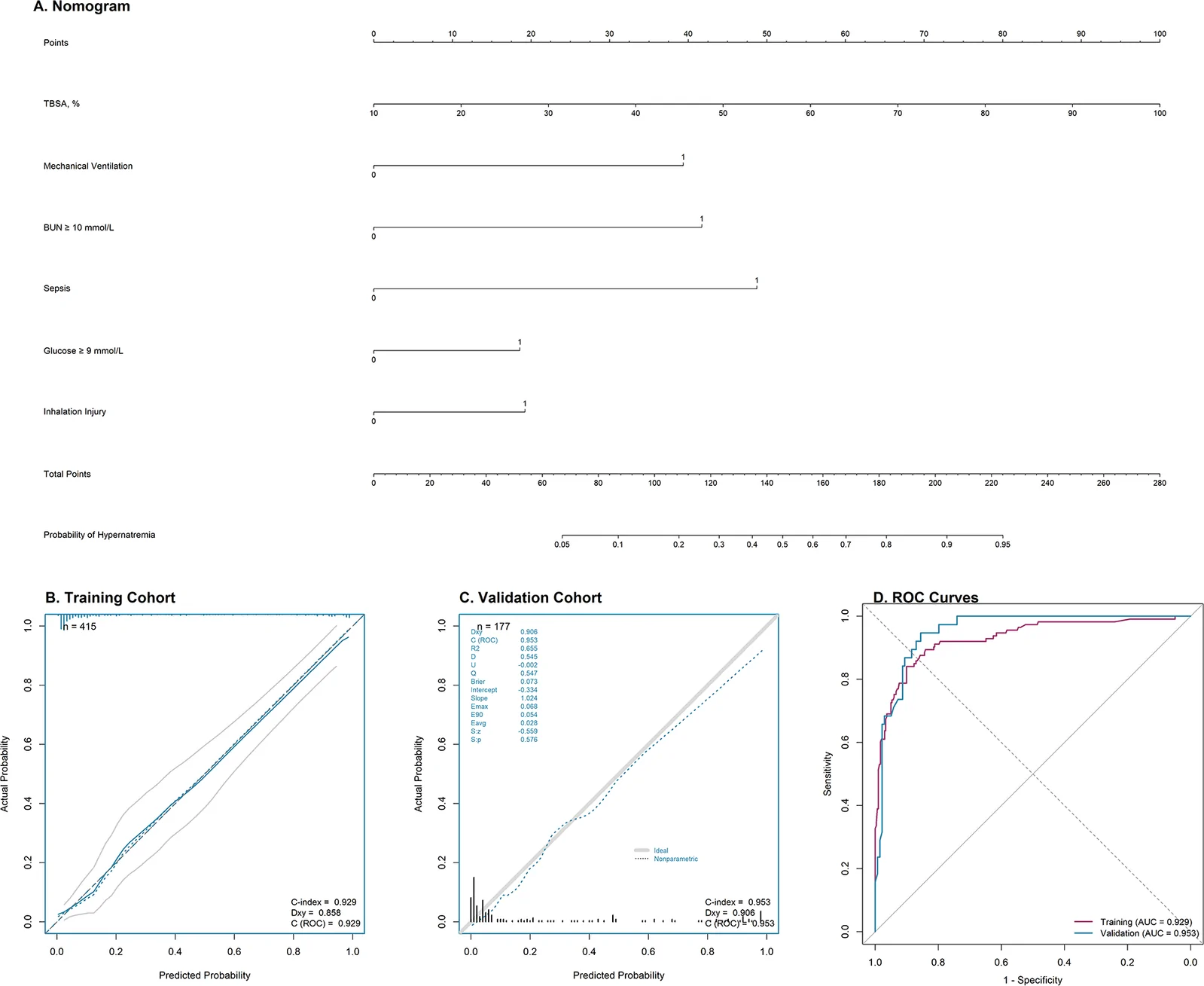
Nomogram and predictive performance. **(A)** Nomogram for estimating the risk of hypernatremia in patients with severe burns. To use the nomogram, locate the value of each variable on its corresponding axis, draw a vertical line up to the “Points” axis to determine the number of points for each variable, sum the points for all variables, and then project this total from the “Total Points” axis down to the bottom scale to obtain the predicted probability of hypernatremia. **(B)** Calibration of the nomogram in the training cohort (*n* = 415). **(C)** Calibration of the nomogram in the internal validation cohort (*n* = 177). **(D)** Receiver operating characteristic (ROC) curves of the nomogram in the training and internal validation cohorts. AUC, area under the ROC curve.

**Table 2:** Univariable logistic regression analysis of hypernatremia in the training cohort.

Variable	OR (95%CI)	*P* value
Age, y	1.033 (1.016–1.051)	<.001
Sex, male vs. female	1.201 (0.688–2.099)	.52
TBSA, %	1.069 (1.054–1.083)	<.001
Full-thickness burn area, ＞ 20% vs. ≤20%	6.861 (4.018–11.714)	<.001
IL−6, ＞ 200 vs. ≦200	4.800 (3.001–7.676)	<.001
PCT, ＞ 0.5 vs. ≤0.5	8.008 (4.842–13.244)	<.001
Minimum Albumin, g/L	0.887 (0.846–0.929)	<.001
WBC, ×10⁹/L	1.046 (1.025–1.068)	<.001
NEUT, ×10⁹/L	1.054 (1.031–1.078)	<.001
Mechanical Ventilation, yes vs. no	15.130 (8.910–25.692)	<.001
Airway Incision, yes vs. no	12.290 (7.277–20.757)	<.001
Cr, μmol/L	1.016 (1.011–1.022)	<.001
BUN, mmol/L	1.495 (1.363–1.640)	<.001
Sepsis, yes vs. no	21.352 (9.172–49.710)	<.001
Glucose, mmol/L	1.240 (1.162–1.323)	<.001
Inhalation Injury, yes vs. no	7.647 (4.506–12.976)	<.001
Fluidized Bed, yes vs. no	4.535 (2.869–7.171)	<.001

OR, odds ratio; CI, confidence interval; TBSA, total body surface area; IL−6, interleukin 6; PCT, procalcitonin; WBC, white blood cell count; NEUT, neutrophil count; Cr, creatinine; BUN, blood urea nitrogen.

**Table 3:** Multivariable logistic regression analysis of hypernatremia in the training cohort.

Variable	*β*'	OR (95%CI)	*P* value
TBSA, %	0.042	1.043 (1.025–1.061)	<.001
Mechanical Ventilation, yes vs. no	1.477	4.38 (2.20–8.73)	<.001
BUN, ≥10 vs. <10 mmol/L	1.566	4.79 (2.42–9.49)	<.001
Sepsis, yes vs. no	1.828	6.22 (1.88–20.58)	.003
Glucose, ≥9 vs. <9 mmol/L	0.697	2.01 (1.02–3.96)	.045
Inhalation Injury, yes vs. no	0.722	2.06 (1.02–4.16)	.045

TBSA, total body surface area; BUN, blood urea nitrogen; OR, odds ratio; CI, confidence interval.

The final model was internally validated using bootstrap resampling. The nomogram showed high accuracy in estimating the risk of hypernatremia, with an apparent C-index of 0.929 (95% CI, 0.899–0.959) and a bootstrap-corrected C-index of 0.923. Calibration plots indicated close agreement between predicted risk and observed outcomes (Figures 3B,C), and ROC curves demonstrated excellent discrimination in both the training and internal validation cohorts (Figure 3D). In the internal validation cohort, the nomogram achieved a C-index of 0.953 (95% CI, 0.924–0.982). Ten-fold cross-validation produced a mean C-index of 0.924 (SD, 0.048) (Supplementary Table S2).

### 3.4 Risk stratification based on nomogram scores

Two cutoff scores were derived from ROC curve analysis: a low cutoff of 90 points (corresponding to a predicted probability of approximately 0.10) to rule out low-risk patients, and a high cutoff of 135 points (corresponding to a predicted probability of 0.35) to rule in high-risk patients. Based on these thresholds, patients were categorized into three risk groups: low risk (<90 points), intermediate risk (90–134 points), and high risk (≥135 points). The sensitivity, specificity, PPV, NPV, and likelihood ratios for each cutoff in the training and internal validation cohorts are summarized in Table 4. The NPV at the low cutoff was 96.4% and 98.1% in the training and internal validation cohorts, respectively, supporting the safe exclusion of low-risk patients; the PPV at the high cutoff was 75.4% and 65.3%, respectively, supporting the accurate identification of high-risk patients. Decision-curve analysis showed that the nomogram offered a net clinical benefit across threshold probabilities ranging from 10% to 35% in both the training and internal validation cohorts, compared to the “treat all” and “treat none” strategies (Figure 4). Importantly, this range includes the two cutoff values used for risk stratification—90 and 135 points—which correspond to predicted probabilities of approximately 0.10 and 0.35. This finding suggests that the model is clinically useful within the threshold range most relevant to decision-making.

**Figure 4:**
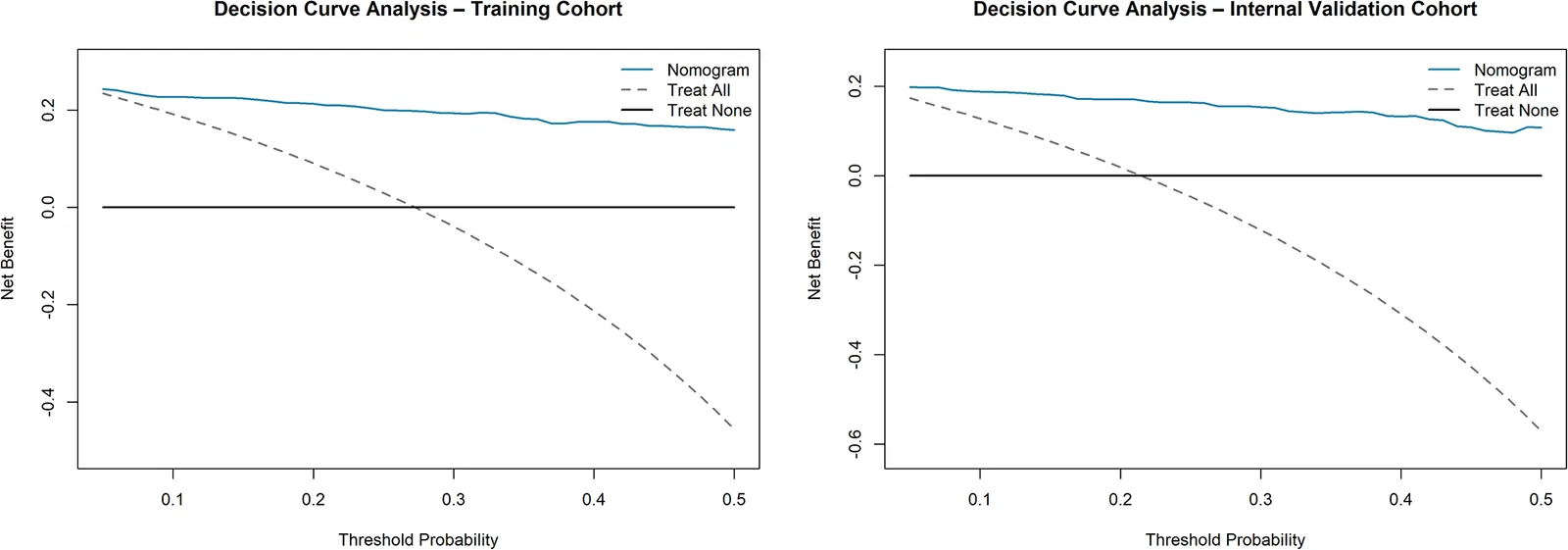
Decision-curve analysis of the nomogram. The *x*-axis represents threshold probability; the *y*-axis represents net benefit. The blue solid line represents the net benefit of using the nomogram to guide clinical decisions; the grey dashed line represents the “treat all” strategy; the horizontal black line at zero represents the “treat none” strategy. The nomogram provided a net clinical benefit across threshold probabilities of approximately 10% to 35% in both cohorts.

**Table 4:** Accuracy of the prediction score of the nomogram.

Variable	Value(95%CI)	
	Training cohort	Validation cohort
Area under ROC curve, C-index	0.929 (0.899–0.959)	0.953 (0.924–0.982)
Low cutoff score (rule-out), total points	90	90
Sensitivity, %	92.9 (86.6–96.4)	94.7 (82.7–98.5)
Specificity, %	71.2 (65.8–76.0)	75.5 (67.8–81.9)
Positive predictive value, %	54.7 (47.6–61.6)	51.4 (40.0–62.8)
Negative predictive value, %	96.4 (93.1–98.2)	98.1 (93.4–99.5)
Positive likelihood ratio	3.23 (2.68–3.88)	3.87 (2.86–5.24)
Negative likelihood ratio	0.10 (0.05–0.19)	0.07 (0.02–0.27)
High cutoff score (rule-in), total points	135	135
Sensitivity, %	78.8 (70.3–85.3)	84.2 (69.6–92.6)
Specificity, %	90.4 (86.5–93.2)	87.8 (81.3–92.2)
Positive predictive value, %	75.4 (66.9–82.3)	65.3 (51.3–77.1)
Negative predictive value, %	91.9 (88.3–94.5)	95.3 (90.2–97.8)
Positive likelihood ratio	8.20 (5.73–11.75)	6.89 (4.32–10.97)
Negative likelihood ratio	0.23 (0.16–0.34)	0.18 (0.09–0.38)

## 4 Discussion

In this study, approximately 27.2% of patients with severe burns developed hypernatremia during hospitalization. The occurrence of hypernatremia was associated with a markedly increased risk of death and a longer ICU length of stay. We also found that several early clinical factors—including larger TBSA, mechanical ventilation, elevated BUN, elevated blood glucose, sepsis, and inhalation injury—were significantly associated with the subsequent development of hypernatremia.

Previous studies have largely relied on single clinical variables or isolated laboratory biomarkers to predict hypernatremia in burn patients, and comprehensive prediction models have been limited (1, 8, 19, 20). In addition, because most of these investigations did not specifically target patients with severe burns, their clinical applicability to this high-risk population has been restricted. Among currently available prediction tools, nomograms combine multiple predictors into a single graphical model, providing high accuracy, strong discrimination, and user-friendly clinical application. In the present study, we developed a nomogram that integrates six easily obtainable early variables. Its performance was strong, as indicated by the C-index values in the training and internal validation cohorts (0.929 and 0.953, respectively), and by calibration curves showing excellent agreement between predicted and observed outcomes. To further assess model robustness, we performed 10-fold cross-validation in the training cohort. The mean cross-validated C-index was 0.924 (SD, 0.048), which closely matched the bootstrap-corrected C-index of 0.923, indicating good internal stability. In addition, the C-index in the internal validation cohort reached 0.953 (95% CI, 0.924–0.982), supporting the model's excellent generalizability. In this study, the C-index was slightly higher in the internal validation cohort than in the training cohort (0.953 vs. 0.929). This difference is likely due to the smaller sample size of the validation cohort (*n* = 177) and its lower event rate (21.5% vs. 27.2%). These factors can lead to wider confidence intervals and occasional overestimation of discrimination. Additionally, random variation from the split-sample design may have influenced this result. Notably, the bootstrap-corrected and cross-validated C-indices in the training cohort were consistent (0.923 and 0.924, respectively). This consistency indicates robust model performance and suggests that the higher C-index in the validation cohort does not reflect a systematic issue in the primary data.

In this nomogram, a larger TBSA indicates more extensive disruption of the skin barrier. As the largest organ in the body, the skin loses its barrier function after severe burns, resulting in markedly increased insensible water loss through the wound surface (21). In our study, the risk of hypernatremia increased by approximately 4.3% for each 1% increase in TBSA (OR, 1.043), consistent with the pathophysiological mechanism whereby evaporative water loss rises in proportion to burn size in patients with extensive burns. The increased risk associated with mechanical ventilation may be explained by several factors. First, patients requiring mechanical ventilation are generally more critically ill and tend to have larger burn areas. Second, insensible water loss through the respiratory tract increases during mechanical ventilation, particularly when humidification is inadequate (22, 23). Third, sedative agents may alter the thirst center and impair regulation of water intake (24). Mapata et al. (25) reported that mechanical ventilation is an independent risk factor for hypernatremia in critically ill patients, which is consistent with our findings. Elevated BUN reflects both renal dysfunction and volume depletion caused by prerenal factors (26). After severe burns, large volumes of fluid shift from the intravascular to the interstitial space, leading to reduced effective circulating blood volume, decreased renal perfusion, and elevated BUN. At the same time, volume depletion stimulates antidiuretic hormone release; however, burn patients often cannot restore water balance because of ongoing evaporative loss from the wound surface and inadequate oral intake, predisposing them to hypernatremia (11). Sepsis, a common complication after severe burns, induces fever, hyperventilation, abnormal fluid distribution, and metabolic disturbances, all of which increase free water loss. The associated systemic inflammatory response further aggravates capillary leakage, complicating fluid management (27). Inhalation injury directly damages the airway mucosa and increases insensible water loss through the respiratory tract. These patients frequently require fluid restriction to limit pulmonary edema, which can conflict with the need for adequate resuscitation and thereby further increase the risk of hypernatremia. Elevated blood glucose causes osmotic diuresis and additional free water loss, constituting another important risk factor (28). Consistently, Li et al. (29) reported detrimental effects of hyperglycemia on fluid and electrolyte balance in burn patients.

In this study, we developed a comprehensive prediction model using a nomogram that incorporates readily obtainable clinical indicators, including burn surface area, mechanical ventilation, BUN, sepsis, inhalation injury, and blood glucose. Although certain biomarkers, such as inflammatory cytokines, have been reported to be associated with electrolyte disturbances (30), their clinical use is constrained by high testing costs and limited availability, particularly in primary healthcare settings. By contrast, all variables in our model are derived from routine clinical practice, providing excellent accessibility and broad generalizability.

For clinical application, we implemented a dual-cutoff strategy to stratify patients into three risk tiers. The negative predictive value (NPV) of the low cutoff score (90 points) was 96.4% and 98.1% in the training and internal validation cohorts, respectively, with negative likelihood ratios of 0.10 and 0.07. These results indicate that patients classified as low risk (<90 points) have a very low probability of developing hypernatremia and may be considered for less intensive monitoring and aggressive fluid restriction, thereby reducing the risk of complications such as renal hypoperfusion due to excessive fluid restriction. The positive predictive value (PPV) of the high cutoff score (135 points) was 75.4% and 65.3% in the training and internal validation cohorts, respectively, with positive likelihood ratios of 8.20 and 6.89. Thus, the probability of developing hypernatremia in the high-risk group is approximately 7- to 8-fold higher than in the low-risk group. For patients with scores ≥135 points (high risk), clinicians may consider proactive preventive strategies, including preferential use of low-sodium resuscitation fluids, strict glycemic control, aggressive anti-infective therapy, optimization of mechanical ventilation parameters to minimize insensible water loss, and more frequent serum sodium monitoring. Patients with intermediate risk (90–134 points) require closer electrolyte surveillance and individualized fluid management. This three-tier risk stratification strategy offers a more nuanced framework for clinical decision-making. The nomogram developed in this study not only demonstrates high predictive accuracy but also enables risk stratification using dual cutoff values, providing greater clinical utility than a single cutoff approach. Compared with existing scoring systems, the nomogram is displayed in a graphical format that allows clinicians to quickly calculate total scores and estimate risk at the bedside without computer software, facilitating its use across different levels of healthcare institutions. To further improve clinical utility, we converted the nomogram into a web-based interactive calculator. Clinicians can obtain real-time predicted probabilities and risk stratification simply by entering patient parameters into the online interface, without performing any manual calculations. The tool is deployed on a free platform and can be accessed from any device with an internet connection. Hypernatremia risk is only one consideration in burn management. Other important clinical factors—such as patient age, comorbidities, burn depth, anatomical location, changes in renal function, diuretic use, the composition and rate of fluid resuscitation, the route and composition of nutritional support, and the timing of wound excision and closure—are not included in the model and must also inform treatment decisions. These unmeasured variables may help explain the false-positive predictions observed at the high cutoff, as the positive predictive value was 75.4% in the training cohort and 65.3% in the internal validation cohort. Therefore, clinicians should use the nomogram to supplement, rather than replace, their clinical judgment.

Our study has several limitations. First, this analysis was conducted at a single institution, and external validation in other centers is required to confirm the generalizability of our findings. Second, the retrospective design underscores the need for prospective studies to further verify the reliability of the nomogram. Third, we excluded patients whose hospital stay was less than 72 h to allow for adequate assessment of predictors and outcomes. However, this approach may have introduced selection bias by omitting patients who died or experienced rapid deterioration soon after admission. As a result, our findings may not be fully generalizable to this subgroup. Fourth, the model was developed through univariable screening and backward stepwise selection, followed by dichotomizing blood urea nitrogen and glucose using data-derived cutoffs. This method may increase the risk of overfitting and lead to information loss from continuous variables. However, internal validation with bootstrap resampling and 10-fold cross-validation showed minimal optimism. These results suggest that the impact of these methodological choices was limited. Fifth, this study covered a 10-year period, during which clinical practices such as fluid resuscitation protocols and monitoring strategies may have changed. Although our institution consistently used a standardized burn resuscitation protocol based on the modified Parkland formula, we cannot rule out minor shifts in practice patterns or monitoring intensity that could have affected the incidence of hypernatremia. Sixth, the retrospective design limited our ability to determine the exact timing of hypernatremia onset in relation to the development of individual risk factors. While we verified that all predictor variables were assessed during the early post-admission period and before the first recorded episode of hypernatremia, the precise interval between the occurrence of risk factors and the onset of hypernatremia was not available for all patients. Seventh, hypernatremia was evaluated as a binary outcome, defined by the presence or absence of at least two sodium measurements exceeding 145 mmol/L. This dichotomization may have led to a loss of information concerning the severity and duration of sodium elevation, which could introduce residual confounding. Future studies might explore continuous sodium trajectories or time-to-hypernatremia as alternative outcome measures. Eighth, although the nomogram showed good overall predictive performance, the false-positive rate at the low cutoff (90 points) in the internal validation cohort remains approximately 25%, and the false-negative rate at the high cutoff (135 points) in the internal validation cohort remains approximately 35%; these error rates warrant careful consideration if major clinical decisions are to be guided by this tool. Finally, the model's reliance on routine clinical data resulted in the inconsistent recording of several potentially relevant variables. These include the composition and rate of resuscitation fluids, the route and composition of nutritional support, diuretic use, dynamic changes in renal function, and detailed insensible loss management interventions***.*** Consequently, these variables could not be included in the model. Future prospective studies should aim to collect these variables to refine the prediction model further. Additionally, the incorporation of novel hypernatremia-specific biomarkers may enhance its predictive accuracy.

## 5 Conclusions

By integrating six early independent risk factors for hypernatremia, we developed a nomogram and a web-based calculator that together provide an optimized tool for predicting hypernatremia risk in patients with severe burns. This model enables early identification of high-risk and low-risk individuals, thereby supporting the implementation of individualized preventive strategies in clinical practice.

## Data Availability

The datasets analyzed during the current study are not publicly available due to institutional restrictions on patient data sharing but are available from the corresponding author upon reasonable request. The R code for the statistical analysis and the web-based interactive calculator are publicly accessible at https://fmmuliubo.shinyapps.io/hypernatremia_nomogram/.

## References

[1] Sedghiani I Mokline A Fredj H Bouguezzi N Gamara FZ Ben Saad M. Hypernatremia risk factors and prognostic impact in burn patients: a case control study. Ann Burns Fire Disasters. (2021) 34(2):135–9. 34584500 PMC8396159

[2] Gutowska I Żwierełło W Piorun K Skórka-Majewicz M Maciejewska-Markiewicz D Kupnicka P. The extent of burn injury significantly affects serum micro- and macroelement concentrations in patients on the first day of hospitalisation. Nutrients. (2022) 14(20):4248. 10.3390/nu14204248 36296932 PMC9610650

[3] Sen S Tran N Chan B Palmieri TL Greenhalgh DG Cho K. Sodium variability is associated with increased mortality in severe burn injury. Burns Trauma. (2017) 5:34. 10.1186/s41038-017-0098-4 29142896 PMC5674226

[4] White KC Nasser A Chaba A Bellomo R. Hypernatremia of critical illness. J Transl Crit Care Med. (2025) 7(1):e24–39. 10.1097/JTCCM-D-24-00039

[5] Flynn K Hatfield J Brown K Vietor N Hoang T. Central and nephrogenic diabetes insipidus: updates on diagnosis and management. Front Endocrinol (Lausanne). (2024) 15:1479764. 10.3389/fendo.2024.1479764 39845881 PMC11750692

[6] Deetjen P Kaspar M Hinske LC Weiss M Heller AR Simon P. Beneficial effects of a departmental protocol to reduce intensive care hypernatremia: an observational before-and-after study. Minerva Anestesiol. (2026) 92(4):305–15. 10.23736/S0375-9393.25.19484-4 41801115

[7] Liu X Wang Z Zhang Y Luan X. Construction and validation of a LASSO penalized logistic regression model predicting hypernatremia after pituitary adenoma surgery. Sci Rep. (2025) 15(1):38552. 10.1038/s41598-025-22512-2 41188349 PMC12586533

[8] Li L Shen C Sun T Cai J Deng H Li D. The role of systemic inflammatory response, hypermetabolism, and wound treatment and repair in hypernatremia of severely burned patients. Regenesis Repair Rehabil. (2025) 1(2):7–13. 10.1016/j.rerere.2025.02.001

[9] Boutin L Kadri F Chaftar A Deniau B Minani S Figueroa SM. From haemodynamics to kidney risk: AI-based early prediction validated in general and burn ICU populations. Eur Heart J Digit Health. (2026) 7(1):ztaf150. 10.1093/ehjdh/ztaf150 41574040 PMC12822602

[10] Wu Guoqing ZJ. Influencing factors for disease progression in severe burn patients with acute kidney injury. Chin J Nephrol Dial Transplant. (2026) 35(1):22–6.

[11] ArabiDarrehDor G Kramer GC Burmeister DM Salinas J Hahn JO. A mathematical model for simulation of cardiovascular, renal, and hormonal responses to burn injury and resuscitation. Front Physiol. (2024) 15:1467351. 10.3389/fphys.2024.1467351 39421439 PMC11484069

[12] Nelson M. Resuscitation of patients with burns: guidelines from the American burn association. Am Fam Physician. (2025) 112(2):219–20. 40834387

[13] Hettiaratchy S Papini R. Initial management of a major burn: II–assessment and resuscitation. Br Med J. (2004) 329(7457):101–3. 10.1136/bmj.329.7457.101 PMC449823 15242917

[14] Giurgiu RA Bordeanu-Diaconescu EM Grosu-Bularda A Frunza A Grama S Dumitru C. Comprehensive review of clinical presentation, treatment, and prognostic factors of airway burns. J Med Life. (2025) 18(5):405–10. 10.25122/jml-2025-0081 40599137 PMC12207697

[15] Rajaratnam G Baldwin AJ. To BAL or not to BAL, that is the question": variations in smoke inhalation injury guidelines from burn units and centres in England, Scotland and Wales. Burns. (2024) 50(9):107273. 10.1016/j.burns.2024.09.012 39353794

[16] Erickson MJ Enkhbaatar P Lee JO. Inhalation injury. Semin Plast Surg. (2024) 38(2):93–6. 10.1055/s-0044-1782646 38746701 PMC11090654

[17] Ostermann M Lumlertgul N Jeong R See E Joannidis M James M. Acute kidney injury, Lancet. (2025) 405(10474):241–56. 10.1016/S0140-6736(24)02385-7 39826969

[18] Greenhalgh DG Saffle JR Holmes JH Gamelli RL Palmieri TL Horton JW. American Burn Association Consensus conference to define sepsis and infection in burns, J Burn Care Res. (2007) 28(6):776–90. 10.1097/BCR.0B013E3181599bc9 17925660

[19] Menger MM Wenz H Bamberg M Krauß S Lauer H Viergutz T. Severe burn injuries—the day the sodium starts rising. In Vivo. (2024) 38(2):747–53. 10.21873/invivo.13497 38418157 PMC10905458

[20] Stewart IJ Morrow BD Tilley MA Snow BD Gisler C Kramer KW. Dysnatremias and survival in adult burn patients: a retrospective analysis. Am J Nephrol. (2013) 37(1):59–64. 10.1159/000346206 23327805

[21] Arlati S Aseni P. From evaporation to edema: a scoping review of physical and biological determinants of early fluid distribution in burn patients. Eur Burn J. (2026) 7(2):21. 10.3390/ebj7020021 42029575 PMC13108070

[22] Kelly M Gillies D Todd DA Lockwood C. Heated humidification versus heat and moisture exchangers for ventilated adults and children. Cochrane Database Syst Rev. (2010) 4:Cd004711. 10.1002/14651858.CD004711.pub2 20393939

[23] Deokar K Plotnikow GA Singhal S Mina B ElShazly M Wooldridge M. Humidification insights in mechanical ventilation and respiratory support. A narrative review. Expert Rev Respir Med. (2026) 20(4):393–406. 10.1080/17476348.2025.2581342 41160622

[24] Yang W Li H Cheng Z Lu Y Li W Feng J. Dex modulates the balance of water-electrolyte metabolism by depressing the expression of AVP in PVN. Front Pharmacol. (2022) 13:919032. 10.3389/fphar.2022.919032 36081946 PMC9445239

[25] Mapata L Richards GA Laher AE. Hypernatremia at a tertiary hospital intensive care unit in South Africa. Cureus. (2022) 14(2):e22648. 10.7759/cureus.22648 35371787 PMC8962634

[26] Rugg C Ströhle M Schmid S Kreutziger J. The link between hypermetabolism and hypernatremia in severely burned patients. Nutrients. (2020) 12(3):774. 10.3390/nu12030774 32183417 PMC7146173

[27] Lin CY Chen YM Tsai YH Hung KY Fang YT Chang YP. Association of hypernatremia with immune profiles and clinical outcomes in adult intensive care unit patients with sepsis. Biomedicines. (2022) 10(9):2285. 10.3390/biomedicines10092285 36140385 PMC9496274

[28] Wagner B Ing TS Roumelioti ME Sam R Argyropoulos CP Lew SQ. Hypernatremia in hyperglycemia: clinical features and relationship to fractional changes in body water and monovalent cations during its development. J Clin Med. (2024) 13(7):1957. 10.3390/jcm13071957 38610721 PMC11012913

[29] Li Y Liu Y Xie S Zhu Y Ding X Zhang W. Metabolic response to burn injury: a comprehensive bibliometric study. Front Med (Lausanne). (2024) 11:1451371. 10.3389/fmed.2024.1451371 39830385 PMC11739346

[30] Khalaf F Touma D Pappas A Hatim L Wojtowicz-Piotrowski S Jeschke MG. Decoding burn trauma: biomarkers for early diagnosis of burn-induced pathologies. Biomark Res. (2024) 12(1):160. 10.1186/s40364-024-00707-5 39716257 PMC11668120

