# Supplementary material for: Development and validation of a prediction model for hypernatremia in patients with severe burns
Source: Front Med (Lausanne). 2026 Sep 30;13:1963702. doi: 10.3389/fmed.2026.1963702 (PMC13661998; doi:10.3389/fmed.2026.1963702)
Supplement: Supplementary file 1 [file Table1.docx]

|  | Cohort, No. (%) |  |  |
| --- | --- | --- | --- |
| Variable | Without Hypernatremia (n=441) | With Hypernatremia (n=151) | P Value |
| Age, mean (SD), y | 39.1 (13.2) | 45.2 (14.3) | <.001 |
| Sex  Male  Female | 342 (77.6) 99 (22.4) | 122 (80.8) 29 (19.2) | .471 |
| TBSA, mean (SD), % | 49.1 (20.2) | 77.2 (20.2) | <.001 |
| Full-thickness burn area |  |  |  |
| ＞20%  ≤20% | 177 (40.1) 264 (59.9) | 121 (80.1) 30 (19.9) | <.001 |
| IL-6, pg/ml  ＞200  ≦200 | 87 (19.7) 354 (80.3) | 76 (50.3) 75 (49.7) | <.001 |
| PCT, ng/ml  ＞0.5  ≤0.5 | 131 (29.7) 310 (70.3) | 113 (74.8) 38 (25.2) | <.001 |
| Minimum Albumin,  mean (SD), g/L | 27.9 (5.3) | 24.6 (5.6) | <.001 |
| WBC, mean (SD), ×10⁹/L | 19.1 (9.3) | 24.5 (13.6) | <.001 |
| NEUT, mean (SD), ×10⁹/L | 16.2 (8.4) | 21.3 (12.4) | <.001 |
| Mechanical Ventilation  Yes  No | 79 (17.9) 362 (82.1) | 121 (80.1) 30 (19.9) | <.001 |
| Airway Incision  Yes  No | 94 (21.3) 347 (78.7) | 121 (80.1) 30 (19.9) | <.001 |
| Cr, mean (SD), μmol/L | 77.3 (38.8) | 125.6 (98.8) | <.001 |
| BUN, mean (SD), mmol/L | 7.3 (4.1) | 15.5 (8.4) | <.001 |
| Sepsis  Yes  No | 11 (2.5) 430 (97.5) | 51 (33.8) 100 (66.2) | <.001 |
| Glucose, mean (SD), mmol/L | 7.6 (2.9) | 10.5 (5.0) | <.001 |
| Inhalation Injury  Yes  No | 154 (34.9) 287 (65.1) | 123 (81.5) 28 (18.5) | <.001 |
| Fluidized Bed  Yes  No | 112 (25.4) 329 (74.6) | 95 (62.9) 56 (37.1) | <.001 |

**Supplementary Table 1. Comparison of Clinical Characteristics Between Patients With and Without Hypernatremia**
