# Supplementary material for: Development and validation of a prediction model for hypernatremia in patients with severe burns
Source: Front Med (Lausanne). 2026 Sep 30;13:1963702. doi: 10.3389/fmed.2026.1963702 (PMC13661998; doi:10.3389/fmed.2026.1963702)
Supplement: Supplementary file 2 [file Table2.docx]

**Supplementary Table 2.** **10-Fold Cross-Validation C-index in the Training Cohort**

| Fold | C-index |
| --- | --- |
| 1 | 0.981 |
| 2 | 0.960 |
| 3 | 0.894 |
| 4 | 0.849 |
| 5 | 0.879 |
| 6 | 0.946 |
| 7  8  9  10  Mean(SD) | 0.907  1.000  0.930  0.890  0.924(0.048) |

Abbreviation: SD, standard deviation. The 10-fold cross-validation was performed in the training cohort (n = 415). In each fold, the model was fitted on approximately 373 patients and validated on approximately 42 patients. The C-index (area under the receiver operating characteristic curve) was calculated for each fold.
