# Supplementary material for: Development and validation of a prediction model for hypernatremia in patients with severe burns
Source: Front Med (Lausanne). 2026 Sep 30;13:1963702. doi: 10.3389/fmed.2026.1963702 (PMC13661998; doi:10.3389/fmed.2026.1963702)
Supplement: Supplementary file 3 [file Table3.docx]

| Section/Topic | Item No. | Checklist Item | Page/Location |
| --- | --- | --- | --- |
| **Title and Abstract** |  |  |  |
| Title | 1 | Identify the study as developing and/or validating a multivariable prediction model, the target population, and the outcome to be predicted. | Title page |
| Abstract | 2 | Provide a summary of objectives, study design, setting, participants, sample size, predictors, outcome, statistical analysis, results, and conclusions. | Abstract |
| **Introduction** |  |  |  |
| Background and objectives | 3a | Explain the medical context and rationale for developing or validating the multivariable prediction model, including references to existing models. | Introduction, paragraphs 1-3 |
| **Methods**  Source of data  Participants  Outcome  Predictors  Sample size  Missing data  Statistical analysis methods  Risk groups  Development vs. validation  **Results**  Participants  Model development  Model specification  Model performance  Model-updating  **Discussion**  Limitations  Interpretation  Implications  **Other Information**  Supplementary information  Funding | 3b  4a  4b  5a  5b  5c  6a  6b  7a  7b  8  9  10a  10b  10c  10d  10e  11  12  13a  13b  13c  14a  14b  15a  15b  16  17  18  19a  19b  20  21  22 | Specify the objectives, including whether the study describes the development and/or validation of the model  Describe the study design or source of data (e.g., randomized trial, cohort, or registry data), separately for the development and validation data sets, if applicable.  Specify the key study dates, including start of accrual; end of accrual; and, if applicable, end of follow-up.  Specify key elements of the study setting (e.g., primary care, secondary care, general population) including number and location of centers.  Describe eligibility criteria for participants.  Give details of treatments received, if relevant.  Clearly define the outcome that is predicted by the prediction model, including how and when assessed.  Report any actions to blind assessment of the outcome to be predicted.  Clearly define all predictors used in developing or validating the multivariable prediction model, including how and when they were measured.  Report any actions to blind assessment of predictors for the outcome and other predictors.  Explain how the study size was arrived at.  Describe how missing data were handled (e.g., complete-case analysis, single imputation, multiple imputation) with details of any imputation method.  Describe how predictors were handled in the analyses.  Specify type of model, all model-building procedures (including any predictor selection), and method for internal validation.  For validation, describe how the predictions were calculated.  Specify all measures used to assess model performance and, if relevant, to compare multiple models.  Describe any model updating (e.g., recalibration) arising from the validation, if done.  Provide details on how risk groups were created, if done.  For validation, identify any differences from the development data in setting, eligibility criteria, outcome, and predictors.  Describe the flow of participants through the study, including the number of participants with and without the outcome and, if applicable, a summary of the follow-up time. A diagram may be helpful.  Describe the characteristics of the participants (basic demographics, clinical features, available predictors), including the number of participants with missing data for predictors and outcome.  For validation, show a comparison with the development data of the distribution of important variables (demographics, predictors, and outcome).  Specify the number of participants and outcome events in each analysis.  If done, report the unadjusted association between each candidate predictor and outcome.  Present the full prediction model to allow predictions for individuals (i.e., all regression coefficients, and model intercept or baseline survival at a given time point).  Explain how to use the prediction model.  Report performance measures (with CIs) for the prediction model.  If done, report the results from any model updating (i.e., model specification, model performance).  Discuss any limitations of the study (such as nonrepresentative sample, few events per predictor, missing data).  For validation, discuss the results with reference to performance in the development data, and any other validation data.  Give an overall interpretation of the results, considering objectives, limitations, results from similar studies, and other relevant evidence.  Discuss the potential clinical use of the model and implications for future research.  Provide information about the availability of supplementary resources, such as study protocol, Web calculator, and data sets.  Give the source of funding and the role of the funders for the present study. | Introduction, last paragraph  Methods, Study Population, paragraph 1  Methods, Study Population, paragraph 1; Results, Prognostic Implications, paragraph 1  Methods, Study Population, paragraph 1  Methods, Study Population, paragraph 2  Methods, Clinical Management and Data Collection, paragraph 1  Methods, Clinical Management and Data Collection, paragraph 2  Not applicable (retrospective study)  Methods, Clinical Management and Data Collection, paragraph 2; Table 1  Not applicable (retrospective study)  Methods, Study Population, paragraph 2; Figure 1  Methods, Study Population, paragraph 2 (exclusion criteria: >30% missing); Statistical Analysis, paragraph 2 (complete-case analysis)  Methods, Statistical Analysis, paragraph 2  Methods, Statistical Analysis, paragraphs 2-3  Methods, Statistical Analysis, paragraph 3  Methods, Statistical Analysis, paragraph 3 (C-index, calibration plots, bootstrap, cross-validation)  Not done  Methods, Statistical Analysis, paragraph 4  Methods, Study Population, paragraph 2; Table 1  Results, Clinicopathologic Characteristics, paragraph 1; Figure 1; Table 1  Table 1  Table 1 (P values comparing cohorts)  Table 3 legend (n=415, events=113)  Table 2  Table 3; Figure 3A  Figure 3 legend; Results, Risk Stratification; online calculator URL  Results, Development and Validation paragraphs 3-4;  Not done  Discussion, last paragraph  Discussion, paragraphs 1-3  Discussion  Discussion, paragraphs 4-5  Discussion (URL provided); Data Availability statement; Supplementary Materials  Acknowledgments (Funding) |
